# Improving outcomes for caregivers through treatment of young people affected by war: a randomized controlled trial in Sierra Leone

**DOI:** 10.2471/BLT.14.139105

**Published:** 2015-10-16

**Authors:** Ryan K McBain, Carmel Salhi, Katrina Hann, Jim Kellie, Alimamy Kamara, Joshua A Salomon, Jane J Kim, Theresa S Betancourt

**Affiliations:** a Department of Global Health and Population, Harvard School of Public Health, 677 Huntington Ave, 7th Floor, Boston, 02115, MA, United States of America (USA).; b FXB Center for Health and Human Rights, Harvard University, Boston, USA.; c CARITAS Freetown, Freetown, Sierra Leone.; d Department of Health Policy and Management, Harvard School of Public Health, Boston, USA.

## Abstract

**Objective:**

To measure the benefits to household caregivers of a psychotherapeutic intervention for adolescents and young adults living in a war-affected area.

**Methods:**

Between July 2012 and July 2013, we carried out a randomized controlled trial of the Youth Readiness Intervention – a cognitive–behavioural intervention for war-affected young people who exhibit depressive and anxiety symptoms and conduct problems – in Freetown, Sierra Leone. Overall, 436 participants aged 15–24 years were randomized to receive the intervention (*n* = 222) or care as usual (*n* = 214). Household caregivers for the participants in the intervention arm (*n* = 101) or control arm (*n* = 103) were interviewed during a baseline survey and again, if available (*n* = 155), 12 weeks later in a follow-up survey. We used a burden assessment scale to evaluate the burden of care placed on caregivers in terms of emotional distress and functional impairment. The caregivers’ mental health – i.e. internalizing, externalizing and prosocial behaviour – was evaluated using the Oxford Measure of Psychosocial Adjustment. Difference-in-differences multiple regression analyses were used, within an intention-to-treat framework, to estimate the treatment effects.

**Findings:**

Compared with the caregivers of participants of the control group, the caregivers of participants of the intervention group reported greater reductions in emotional distress (scale difference: 0.252; 95% confidence interval, CI: 0.026–0.4782) and greater improvements in prosocial behaviour (scale difference: 0.249; 95% CI: 0.012–0.486) between the two surveys.

**Conclusion:**

A psychotherapeutic intervention for war-affected young people can improve the mental health of their caregivers.

## Résumé

**Objectif:**

Mesurer les avantages pour les aidants familiaux d'une intervention psychothérapeutique destinée aux adolescents et aux jeunes adultes qui vivent dans une région touchée par la guerre.

**Méthodes:**

Entre juillet 2012 et juillet 2013, nous avons réalisé à Freetown, en Sierra Leone, un essai contrôlé randomisé de la Youth Readiness Intervention – une intervention cognitivo-comportementale destinée aux jeunes touchés par la guerre qui présentent des symptômes de dépression et d'anxiété ainsi que des troubles du comportement. Au total, 436 participants âgés de 15 à 24 ans ont été sélectionnés de manière aléatoire pour bénéficier soit de l'intervention (*n* = 222), soit d'une prise en charge standard (*n* = 214). Les aidants familiaux des participants du groupe expérimental (*n* = 101) ou du groupe de contrôle (*n* = 103) ont été interrogés à l'occasion d'une étude de base, puis ceux qui étaient disponibles (*n* = 155) l'ont de nouveau été 12 semaines plus tard dans le cadre d'une étude de suivi. Nous avons utilisé une échelle d'évaluation pour estimer la charge qui pèse sur les aidants familiaux en matière de soins liés à la détresse psychique et à la déficience fonctionnelle. La santé mentale – c'est-à-dire l'intériorisation, l'extériorisation et le comportement prosocial – des aidants familiaux a été évaluée à l'aide de la Oxford Measure of Psychosocial Adjustment. Des analyses par régression multiple de l'écart des différences ont été utilisées, dans le cadre d'une intention de traiter, afin d'estimer les effets du traitement.

**Résultats:**

Comparés aux aidants familiaux des participants du groupe de contrôle, les aidants familiaux des participants du groupe expérimental ont fait part d'une réduction plus importante de la détresse psychique (différence d'échelle: 0,252; intervalle de confiance, IC, à 95%: 0,026-0,4782) et d'améliorations plus significatives en matière de comportement prosocial (différence d'échelle: 0,249; IC à 95%: 0.012-0.486) entre les deux études.

**Conclusion:**

Les interventions psychothérapeutiques destinées aux jeunes touchés par la guerre peuvent améliorer la santé mentale des aidants familiaux.

## Resumen

**Objetivo:**

Medir los beneficios para los cuidadores del hogar de una intervención psicoterapéutica para adolescentes y adultos jóvenes que viven en una zona afectada por la guerra.

**Métodos:**

Entre julio de 2012 y julio de 2013, se llevaron a cabo ensayos controlados aleatorizados de la Youth Readiness Intervention (una intervención cognitivo-conductual para jóvenes afectados por la guerra que presentan síntomas de depresión y ansiedad y problemas de conducta) en Freetown, Sierra Leona. En términos generales, 436 participantes de 15 a 24 años fueron aleatorizados para recibir la intervención (*n* = 222) o los cuidados habituales (*n* = 214). Se entrevistó a los cuidadores del hogar de los participantes en el grupo de intervención (*n* = 101) o el grupo de control (*n* = 103) durante un estudio de referencia y de nuevo, si estaban disponibles (*n* = 155), 12 semanas después en un estudio de seguimiento. Se utilizó una escala de valoración de la carga para así evaluar la carga de los cuidados de los cuidadores en términos de angustia emocional y discapacidad funcional. Se evaluó la salud mental de los cuidadores, es decir, el comportamiento de internalización, externalización y prosocial, mediante el uso de la medida de ajuste psicosocial de Oxford. Los análisis de diferencias en diferencias basados en regresiones múltiples se utilizaron, dentro de un marco de intención de tratar, para estimar los efectos del tratamiento.

**Resultados:**

En comparación con los cuidadores de los participantes del grupo de control, los cuidadores de los participantes del grupo de intervención registraron una mayor reducción en la angustia emocional (diferencia en la escala: 0,252; intervalo de confianza (IC) del 95%: 0,026–0,4782) y mayores mejoras en el comportamiento prosocial (diferencia en la escala: 0,249 (IC del 95%: 0,012–0,486) entre los dos estudios.

**Conclusión:**

Una intervención psicoterapéutica para los jóvenes afectados por la guerra puede mejorar la salud mental de sus cuidadores.

## ملخص

**الغرض:**

قياس الفوائد التي تعود على مقدمي الرعاية في المنزل من خلال التدخل بالعلاج النفسي للمراهقين والشباب الذين يعيشون في منطقة متضررة من الحروب.

**الطريقة:**

في الفترة ما بين يوليو من عام 2012 ويوليو من عام 2013، أجرينا تجربة معشاة مضبطة بالشواهد لجاهزية الشباب بالتدخل – وهو تدخل قائم على الإدراك والسلوك للشباب المتأثرين بالحروب والذين يظهرون أعراض الاكتئاب والقلق ويثيرون مشاكل سلوكية - في فريتاون بسيراليون. وبوجه عام، تم اختيار 436 مشاركًا تتراوح أعمارهم من 15 إلى 24 سنة على نحو عشوائي لتلقي التدخل (العدد = 222) أو تلقي الرعاية المعتادة (العدد = 214). وقد أُجريت مقابلات مع مقدمي الرعاية في المنزل للمشاركين في الفرع الخاص بالتدخل (العدد = 101) أو الفرع الخاص بالمجموعة الشاهدة (العدد = 103) خلال مسح خط الأساس ثم تمت إعادة المسح مرة أخرى في حال تواجدهم (العدد = 155)، بعد مرور 12 أسبوعًا خلال مسح المتابعة. استخدمنا مقياس تقدير العبء لتقييم عبء الرعاية الذي وُضع على مقدمي الرعاية من حيث الضائقة الانفعالية والاختلال الوظيفي. وقد تم تقييم الصحة النفسية لمقدمي الرعاية – أي الاستبطان، والاختراج، والسلوك الاجتماعي الإيجابي – باستخدام مقياس أكسفورد للتوافق النفسي. تم استخدام تحاليل متعددة التحوف للاختلافات في الفارق ضمن إطار العمل لقصد العلاج، لتقدير تأثيرات العلاج.

**النتائج:**

بالمقارنة مع مقدمي الرعاية للمشاركين في المجموعة الشاهدة، قام مقدمو الرعاية للمشاركين في مجموعة التدخل بالإبلاغ عن حالات انخفاض أكبر في الضائقة الانفعالية (الفرق في المقياس: 0.252؛ بنسبة أرجحية مقدارها 95%: 0.026–0.4782) وحالات تحسن أكبر في السلوك الاجتماعي الإيجابي (الفرق في المقياس: 0.249؛ بنسبة أرجحية مقدارها 95%: 0.012–0.486) بين المسحين.

**الاستنتاج:**

إن التدخل بالعلاج النفسي للشباب المتأثرين بالحروب يمكنه تحسين الصحة النفسية لمقدمي الرعاية الذين يتولون رعايتهم.

## 摘要

**目的:**

旨在衡量家庭看护人从为居住在战争受灾区的青少年和青壮年提供心理治疗干预中获得的好处。

**方法:**

2012 年 7 月至 2013 年 7 月期间，我们针对塞拉利昂弗里敦市内受战争影响的年轻人（其显示出抑郁和焦虑症状并制造问题）展开了一次青年准备干预（即：认知-行为干预）的随机对照试验。总之，我们随机分配 436 位实验者（15-24 岁）接受干预治疗 (*n* = 222) 或常规护理 (*n* = 214)。基础调查期间，我们采访了接受干预治疗实验者 (*n* = 101) 和接受常规护理实验者的家庭护工 (*n* = 103)，此外，如果可以(*n* = 155)，12 周后在后续调查中继续采访。我们采用负担评估量表评估看护人在情绪抑郁和功能损伤方面肩负的重担。同时使用牛津心理调节衡量措施 (Oxford Measure of Psychosocial Adjustment) 评估看护人的心理健康（即内在行为、外在行为和亲社会行为）。我们在意向治疗范围内，使用差异中之差异重回归分析评估治疗效果。

**结果:**

两次调查之间，相对于对照组实验者的看护人，干预组实验者看护人报告其抑郁情绪减少的人数更多（比例差异：0.252，95% 置信区间 (CI)：0.026–0.4782），而且其亲社会行为大大增强（比例差异：0.249，95% 置信区间 (CI)：0.012–0.486）。

**结论:**

对受战争影响的年轻人进行心理治疗干预可改善其看护人的心理健康。

## Резюме

**Цель:**

Измерить положительное влияние психотерапевтического вмешательства, примененного в лечении подростков и молодых людей, которые проживают в регионах, затронутых войной, на состояние лиц, осуществляющих уход на дому.

**Методы:**

В период с июля 2012 г. по июль 2013 г. было проведено рандомизированное контролируемое исследование программы психосоциального вмешательства в лечении молодых людей (YRI) — когнитивно-поведенческой терапии пострадавших от войны молодых людей, у которых были выявлены симптомы депрессии и тревожного расстройства и отклонения в поведении; исследование проводилось в г. Фритаун, Сьерра-Леоне. Всего 436 участников в возрасте от 15 до 24 лет были распределены случайным образом либо в группу, в которой применялось вмешательство (*n* = 222), либо в группу, в которой уход осуществлялся стандартным способом (*n* = 214). Лица, осуществляющие уход за участниками экспериментальной группы (*n* = 101) или контрольной группы (*n* = 103) на дому, были опрошены в ходе первоначального исследования и по возможности повторно (*n* = 155) через 12 недель в ходе дополнительного исследования. Для наглядности в плане эмоционального расстройства и функциональных нарушений была использована шкала оценки для определения бремени забот, возложенного на лиц, осуществляющих уход. Психическое здоровье лиц, осуществляющих уход, т. е. интернальное, экстернальное и просоциальное поведение, было подвергнуто оценке с применением Оксфордского критерия психосоциальной адаптации. Для оценки результатов лечения был применен метод «разность разностей» на основе множественной линейной регрессии в рамках статистического анализа всех рандомизированных пациентов.

**Результаты:**

По сравнению с лицами, осуществляющими уход за участниками контрольной группы, у лиц, осуществляющих уход за участниками экспериментальной группы, наблюдалось более существенное снижение показателей эмоционального расстройства (разность шкал: 0,252; 95% доверительный интервал, ДИ: 0,026–0,4782) и более существенное улучшение просоциального поведения (разность шкал: 0,249; 95% ДИ: 0,012–0,486) в период между двумя исследованиями.

**Вывод:**

Психотерапевтическое вмешательство в лечении молодых людей, пострадавших от войны, может положительно повлиять на психическое здоровье лиц, осуществляющих уход за ними.

## Introduction

Although, globally, psychiatric disorders account for a larger disease burden than human immunodeficiency virus and malaria combined,^1^ more than three-quarters of individuals living in low- and middle-income countries who have such disorders receive no clinical treatment.^2^ Treatment coverage is particularly poor among children and adolescents with psychiatric disorders^3^ – especially in war-affected countries where displacement, bereavement, the witnessing of violence and limited educational and economic opportunities all contribute to a high prevalence of psychiatric conditions.^4^^–^^7^

There have been few attempts to evaluate the effectiveness of psychotherapeutic interventions among young people affected by war but such interventions appear to have a favourable impact on depressive symptoms^8^ and post-traumatic stress reactions.^9^ It remains unclear if such interventions also lead to indirect benefits among the household caregivers or other household members of the young people. If such side-benefits do exist, the effectiveness of psychotherapeutic interventions for young people may have been underestimated.

Many interventions once thought to have only a focused impact have subsequently been found to have a wider social benefit. Examples include the herd immunity associated with vaccination campaigns^10^ and the spread of healthy behaviour through social networks.^11^ In the context of interventions to improve the lives of war-affected young people, it is possible that improvements in the mental health of the participants translate into enriched household relations and fewer caregiver responsibilities.

There is growing evidence of the transmission of mental health problems, through social networks, from parents to children^12^^,^^13^ and between intimate partners^14^^,^^15^ or siblings.^16^^,^^17^ In France, psychiatric illness has been found to have detrimental effects on employment and overall quality of life of members of the affected person’s household.^18^ Similar qualitative observations have been made in Botswana,^19^ Nigeria^20^ and South Africa.^21^

Here we investigated whether a similar household-level burden of care exists in post-conflict Sierra Leone and whether this burden could be reduced via a cognitive–behavioural intervention for war-affected young people.

## Methods

We did a randomized controlled trial of the Youth Readiness Intervention in young residents of Freetown, Sierra Leone, who had psychological distress and functional impairments. We interviewed caregivers of the young people enrolled in the trial to assess if there was a spill-over effect.

### Intervention

The Youth Readiness Intervention has been described elsewhere.^22^ Briefly, the intervention is a group-based cognitive–behavioural intervention for war-affected young people who show psychological distress and functional impairments as the result of behavioural and emotional problems. To accommodate for multiple problems previously documented among young people in our study area,^23^^,^^24^ we used practice elements of cognitive–behavioural therapy shown to have efficacy across a range of diagnostic categories. Content modules included psychoeducation about trauma, self-regulation and relaxation skills, cognitive restructuring, behavioural activation, communication and interpersonal skills and sequential problem solving.^25^^–^^27^ Given concerns about the safety of addressing complex trauma in a group context^28^ and the limited time we had available to provide specialized training on trauma processing, the intervention did not focus directly on the processing of traumatic memories.

We trained four counsellors for two weeks.^22^ The trained counsellors then led supervised training workshops for other potential counsellors. We employed the eight individuals – four women and four men with bachelor degrees – who completed the training and achieved a high level of competency. A senior local mental health worker provided weekly supervision to all counsellors in-country and the study leaders provided additional clinical supervision by telephone. A local implementation partner, CARITAS Freetown, led the recruitment and management of the study personnel within Freetown.

The intervention took place at six community centres scattered throughout the Western Area of Freetown. In total, there were 26 treatment groups, with a mean of nine participants per group. The groups were separated by sex and age. The interventions groups were led by two trained counsellors of the same sex as the group. Group sessions were held for two hours once a week, over a 10-week period beginning in July 2012.

All of the documents used, including the overarching treatment manual, were translated from English to the local language Krio. The quality of translation was ensured using forward and backward translation under a standardized protocol.^29^

#### Controls

The participants in the control arm received the pre-existing level of care – i.e. the opportunity to self-seek care from the relevant community outreach and youth programmes. Most of the relevant programmes are provided by the Community Association for Psychosocial Services, the organization City of Rest, and Kissy hospital – the only psychiatric hospital in Sierra Leone. Each such programme provides a discrete and relatively narrow set of services. We assumed that, during our trial, few if any of the young people in the control arm sought treatment. All of the participants in the control arm were offered the intervention at the end of the trial.

#### Recruitment and randomization

The sampling frame was a list of Sierra Leonean adolescents and young adults who lived in the study area and were believed to be suffering from a psychiatric disorder. This list was produced after asking community elders, youth leaders at churches and mosques and representatives of local nongovernmental organizations about young people who had been struggling with emotional and behavioural problems. In total, 761 adolescents and young people were screened for eligibility and 436 were enrolled. To be eligible, an individual had to be aged 15 to 24 years, score at least half a standard deviation (SD) above the mean value for internalizing and externalizing symptoms previously documented among war-affected young people in the study area, report impairment in day-to-day functioning and be out of school at the time the trial began.^22^ School might have afforded an informal means of social support that acted as a confounder over the course of the trial. Two individuals were excluded from participation – and referred for individual psychiatric care – because they reported suicidal ideation or symptoms of psychosis.

A randomization sequence generated in Stata version 12.0 SE (StataCorp. LP, College Station, United States of America)^24^ was used to assign participants to the intervention or control arm, with stratification by age group – 15 to 17 or 18 to 24 years – and sex. Randomization occurred after a baseline survey. In total, 222 and 214 participants were assigned to the treatment and control arms, respectively. All 436 participants were interviewed in the baseline survey and again in a follow-up survey that was conducted 12 weeks later, after completion of the intervention. The results of these interviews are reported elsewhere.^30^

### Caregivers

Following randomization of the participants, 204 households were randomly selected for household interviews, 101 in the intervention arm and 103 in the control arm. The participants that belonged to these study households were each asked to identify their primary household caregiver – i.e. the household member primarily responsible for looking after the participant’s well-being. Any participant who was unable to identify such a member was asked to identify the person they were emotionally closest to within their household – under the assumption that this member would be the most invested in the well-being of the participant and therefore could be considered the participant’s caregiver. All caregivers in the study households were interviewed by a trained research assistant who used a structured questionnaire, at baseline and again, if they could be traced, 12 weeks later.

All interviews took place in Krio. None of the caregivers who were interviewed received any components of the intervention. No household members who were invited to participate declined. Interviews typically took one hour to complete and, as an incentive, each interviewee was given a meal.

We used a burden assessment scale^31^ to measure the burden of care placed on the household members of the participants. The four-point ordinal scale takes disrupted activities, personal distress and guilt into account and to verify its cultural relevance we cognitively tested it with the local research team. We did an exploratory factor analysis^32^ to see how the scale performed in our study area. The number of factors was determined by visual inspection of the point of inflexion on the resulting scree plot and by a parallel analysis using 100 000 Monte Carlo simulations.^33^ Several components of the scale performed poorly and we only used the scale to assess forms of emotional distress (9 items) and functional impairment (6 items).

We also assessed three dimensional constructs of mental health – internalizing, externalizing and prosocial behaviour – using the Oxford Measure of Psychosocial Adjustment.^34^ Each item on the measure is evaluated on a four-point ordinal scale that indicates whether the item occurs never, rarely, sometimes or always. Internalizing encompasses depressive and anxiety symptoms, externalizing encompasses aggression and hostility and prosocial behaviour involves actions that demonstrate consideration for the well-being of others.

### Statistical analyses

Based on available resources, the study was powered to detect a between-group standardized mean difference in household-level outcomes of 0.40, given a power of 80% and a serial correlation of 0.50. Our target sample size was 198 caregivers.

We used a difference-in-differences multiple linear regression framework^35^ to compare the magnitude of the changes recorded, between the baseline and follow-up surveys, among the caregivers of the participants in the treatment arm with those recorded among the caregivers of the participants in the control arm – represented as the time × treatment-arm interactions. Potential departures from linearity were examined by plotting standardized residuals against predictor variables. Caregiver age and sex were included as covariates in models, as these main effects were of themselves of interest. For example, we were interested to know if there were baseline differences in mental health between male and female caregivers.

The primary mode of analysis was intention-to-treat – i.e. a caregivers’ data were included even if the associated participant was randomized to receive the intervention but attended no group sessions.

To address missing values in the follow-up data, we used multiple imputation analysis with 100 simulated data sets.^36^^,^^37^ Analyses were conducted in Stata version 12.0 SE

### Ethics

The trial protocol was approved by the Harvard School of Public Health Institutional Review Board as well as an ethics board directed by the Sierra Leonean Ministry of Health. A community advisory board in Freetown also met on a monthly basis to review the trial’s content and implementation. The trial was registered at Clinicaltrials.gov, as RPCGA-YRI-21003. The interviewees – most of whom were illiterate – were asked to provide oral informed consent. To safeguard an interviewee’s privacy, interviewers were trained to identify a location within the interviewee’s house or broader community in which they could conduct the interview privately. As part of the informed consent procedure, participants were informed that they did not have to disclose any information they felt uncomfortable about providing. Participants were also informed that another member of their household may be asked to complete a separate survey.

## Results

### Baseline characteristics

**Fig. 1:**
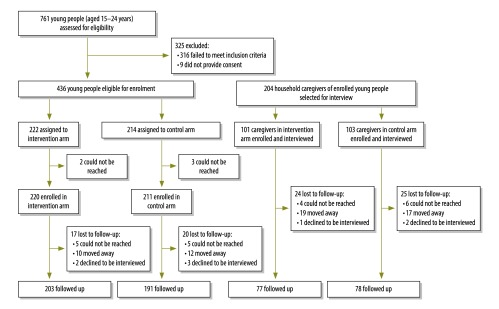
Flowchart to assess the benefit for caregivers of young people enrolled in the Youth Readiness Intervention, Sierra Leone, 2012–2013

Fig. 1 presents a flowchart of the trial. Although 204 caregivers – 97 male and 107 female, with a mean age of 42.3 years (SD: 12.7) and a mean of 3.4 years (SD: 4.0) in education –were interviewed at baseline (Table 1), only 155 (76%) of them were traced and available for the follow-up interviews. Loss to follow-up was the same in both arms.

**Table 1:** Study sample characteristics at baseline, Sierra Leone, 2012

Characteristic	No. (%)	
	Young people^a^ (*n* = 436)	Caregivers (*n* = 204)
**Demographic**		
Female	199 (45.6)	107 (52.5)
Literate	207 (47.7)	76 (37.8)
**War exposure**^b^		
Displaced as result of war	176 (40.4)	150 (73.5)
Friend or family member died due to war	131 (31.1)	148 (73.3)
Witnessed violence or armed conflict	73 (19.7)	98 (48.0)
Direct victim of violence	57 (15.2)	34 (16.8)

^a^ Aged 15 to 24 years.

^b^ Questions about war exposure were not always answered by interviewees.

Most of caregivers were aunts, grandparents, parents, spouses, siblings or uncles of one of the participants (Table 2). At baseline, most of the caregivers reported displacement due to war and the loss of at least one family member or friend due to war (Table 1). The mean age, sex and war exposure were similar between the caregivers in both arms.

**Table 2:** Relationship between the young people investigated and the household caregivers who were interviewed at baseline, Sierra Leone, 2012

Relationship of caregiver to young person	No. of young people^a^ (%)	
	Control arm (*n* = 103)	Intervention arm (*n* = 101)
Mother	23 (22.3)	28 (27.7)
Father	8 (7.8)	13 (12.9)
Sibling	18 (17.5)	10 (9.9)
Aunt or uncle	17 (16.5)	18 (17.8)
Grandparent	8 (7.8)	5 (5.0)
Guardian acting as parent	13 (12.6)	10 (9.9)
Spouse	1 (1.0)	1 (1.0)
Other relative	2 (1.9)	3 (3.0)
Non-relative	5 (4.9)	8 (7.9)
Unknown	8 (7.8)	5 (5.0)

^a^ Aged 15 to 24 years.

Note: Inconsistencies arise in some values due to rounding.

The participants in the two arms were also similar in terms of age, sex and other demographic characteristics. At baseline, the participants had a mean age of 18.0 years (SD: 2.4) and had spent a mean of 8.5 years (SD: 2.1) in school.

### Symptom severity

Table 3 provides an overview of symptom severity among the interviewed caregivers at baseline and follow-up. Although the values missing from the follow-up data were imputed, our main results were nearly identical with and without this imputation procedure and use of the procedure had no effect on the statistical significance of these results.

**Table 3:** Mental health among the caregivers interviewed at baseline and follow-up, Sierra Leone, 2012–2013

Scale, outcome	Potential scores	Mean scores for intervention arm (SD)				Mean scores for control arm (SD)		
		Baseline	Follow-up	Difference		Baseline	Follow-up	Difference
**BAS**^a^								
Emotional distress	1 to 4	1.561 (0.525)	1.359 (0.495)	−0.202		1.396 (0.436)	1.451 (0.545)	0.055
Functional impairment	1 to 4	1.808 (0.566)	1.675 (0.558)	−0.132		1.855 (0.502)	1.666 (0.550)	−0.190
**OMPA**^b^								
Prosocial behaviour	0 to 3	2.268 (0.582)	2.423 (0.544)	0.155		2.235 (0.532)	2.159 (0.539)	−0.076
Internalizing	0 to 3	0.673 (0.387)	0.682 (0.375)	0.009		0.763 (0.434)	0.759 (0.453)	−0.003
Externalizing	0 to 3	0.262 (0.354)	0.319 (0.353)	0.057		0.344 (0.383)	0.408 (0.389)	0.064

BAS: burden assessment scale; OMPA: Oxford Measure of Psychosocial Adjustment; SD: standard deviation.

^a^ Nine and six items were considered in the assessment of emotional distress and functional impairment, respectively.

^b^ Seven items were considered in the assessment of each outcome.

The intervention effects on caregivers are summarized in Table 4. At follow-up, the caregivers of participants given the intervention reported a significantly greater reduction in burden of care – in terms of emotional distress (*P* = 0.03) – than the caregivers of participants assigned to the control arm (associated effect size: 0.51). However, both arms showed similar levels of functional impairment at follow-up (*P* = 0.55). Compared with the caregivers of the participants in the control arm, caregivers of participants given the intervention reported significantly greater improvements in prosocial behaviour (*P* = 0.04; effect size: 0.46) but similar externalizing (*P* = 0.98) and internalizing outcomes (*P* = 0.99).

**Table 4:** Treatment effect of the Youth Readiness Intervention among the caregivers of young people, Sierra Leone, 2012–2013

Outcome	Potential scores	Treatment effect (95% CI)^a^	Effect size^b^
Emotional distress	1 to 4	−0.252 (−0.478 to −0.026)	0.51
Functional impairment	1 to 4	0.073 (−0.169 to 0.315)	0.13
Prosocial behaviour	0 to 3	0.249 (0.012 to 0.486)	0.46
Internalizing	0 to 3	−0.001 (−0.199 to 0.197)	0.00
Externalizing	0 to 3	0.002 (−0.157 to 0.162)	0.00

CI: confidence interval.

^a^ This represents the change recorded in the mean score, between baseline and follow-up, among the caregivers of the young people given the intervention, over and above that observed among the caregivers of the young people in the control arm.

^b^ The effect size of the treatment effect is equivalent to the standardized mean difference.

## Discussion

This intervention was delivered in community-based low-resource settings by local community workers. We found that household caregivers of the trial participants reduced their emotional distress related to burden of care and improved their prosocial behaviour. Since only one member per study household was interviewed, it remains possible that non-interviewed members of the households of participants in the intervention arm also benefitted from the intervention.

While the observed effect sizes might be considered quite modest based on standard metrics, they are relatively large when considered as indirect benefits of an intervention. In terms of the emotional distress scale, for example, the effect size of 0.51 that we observed represents two of the nine questions being scored one ordinal rank lower at follow-up than at baseline. Recent meta-analyses of psychotherapy among children and adolescents in high-income countries indicate that direct benefits to the treated individuals – is represented by an effect size of about 0.30.^38^

Reductions in the level of household caregivers’ emotional distress are notable for several reasons. Most fundamentally, such reductions can improve the caregivers’ overall quality of life in terms of their mental health^39^ and also, in the long term, their physical health,^40^ economic productivity^41^ and civic engagement.^42^ It would be interesting to see if any of the caregivers interviewed in our trial show such long-term benefits. The possibility that the side-benefits of an intervention designed for trial participants support a positive feedback loop within the affected households – i.e. further improvements in the household dynamics and additional benefits to the trial participants and their households – also merits investigation.^43^

There are two processes by which household caregivers of participants who received the intervention are liable to have received the observed benefits. First, a substantial proportion of the intervention’s content focused on the development of actionable skills – e.g. interpersonal, problem-solving and emotion-regulation skills – among the young people. It seems likely that this development had a direct effect on the behaviour of the participants within their households and that this, in turn, improved the household dynamics for all household members. Second, improvements in the emotional state of the young people given the intervention may have had a subtler impact on the other members of their households – i.e. relieving them of some of the guilt and stress they experience.

Compared with the other caregivers interviewed, the caregivers of the participants given the intervention did not demonstrate significantly greater improvements in terms of their functional impairments and externalizing behaviours. Interestingly, functional impairments improved moderately in both of our sets of caregivers. One possible reason for this is that the completion of the intervention coincided with the end of both Ramadan and the rainy season in Sierra Leone – i.e. a period when household members typically spend more time at home and have relatively limited economic opportunities. Thus, the interviewed caregivers may have had fewer responsibilities for the young people at the end of the intervention than at the time of the baseline survey. The relatively low scores for externalizing behaviours at baseline among both sets of caregivers may have hampered measurable improvements.

In the absence of treatment, some war-affected young people with psychiatric disorders have shown worsening outcomes over time^44^ while others have shown improvement.^45^ We sought to recruit participants who had poor psychosocial functioning and who might be unlikely to show any dramatic and stable improvements in the absence of treatment.

Loss to follow-up among caregivers was higher than expected. This was partly due to the highly mobile lifestyles of the study population and partly to challenges in documenting relatively makeshift houses that lack formal addresses. The multiple imputation used to compensate for the missing follow-up data has been shown to be effective for addressing situations in which more than 30% of the potentially useful observations are missing – assuming that observations are missing at random.^46^ This assumption appeared justified in our trial as most of the loss to follow-up that we observed was the consequence of instability in household composition.

Our study had several limitations. We would have to arrange further follow-ups to see if our study trends remained stable over time, became more marked or gradually disappeared in the absence of the intervention. It is possible that, by empowering the young people, the intervention altered household dynamics in ways that we did not investigate. We adapted a burden-of-care scale that had been developed for use in the USA. A more robust approach might entail the development of a new scale specifically for use in Sierra Leone or sub-Saharan Africa as a whole. Given the challenges in finding war-affected young people struggling with mental health problems, we had to rely on a convenience sample.

## Conclusion

The Youth Readiness Intervention appeared to benefit the household caregivers of war-affected young people in two ways: a reduced burden of care – via a reduction in emotional distress – and improved prosocial behaviour. Such side-benefits need to be considered when evaluating the effectiveness and cost–effectiveness of this and similar interventions.
